# Mapping grey matter and cortical thickness alterations associated with subjective cognitive decline and mild cognitive impairment among rural-dwelling older adults in China: A population-based study

**DOI:** 10.1016/j.nicl.2024.103691

**Published:** 2024-10-28

**Authors:** Ziwei Chen, Qianqian Xie, Jiafeng Wang, Yan Wang, Huisi Zhang, Chunyan Li, Yongxiang Wang, Lin Cong, Shi Tang, Tingting Hou, Lin Song, Yifeng Du, Chengxuan Qiu

**Affiliations:** a Key Laboratory of Endocrine Glucose & Lipids Metabolism and Brain Aging, Ministry of Education, Department of Neurology, Shandong Provincial Hospital affiliated to Shandong First Medical University, Jinan, Shandong, PR China; b Department of Neurology, Shandong Provincial Hospital, Shandong University, Jinan, Shandong, PR China; c Shandong Provincial Clinical Research Center for Neurological Diseases, Jinan, Shandong, PR China; d Institute of Brain Science and Brain-Inspired Research, Shandong First Medical University & Shandong Academy of Medical Sciences, Jinan, Shandong, PR China; e Aging Research Center, Department of Neurobiology, Care Sciences and Society, Karolinska Institutet-Stockholm University, Stockholm, Sweden

**Keywords:** Subjective cognitive decline, Mild cognitive impairment, Regional brain volume, Cortical thickness, Magnetic resonance imaging, Population-based study

## Abstract

**Background:**

The structural brain alterations for subjective cognitive decline (SCD) and mild cognitive impairment (MCI) are poorly defined. We sought to characterize grey matter volume (GMV) and cortical thickness associated with SCD and MCI among rural-dwelling older adults in China.

**Methods:**

This population-based cross-sectional study included 1072 dementia-free participants from the brain MRI sub-study of MIND-China (2018–2020). We defined MCI following the Petersen’s criteria, and SCD as the self-rated Ascertain Dementia 8-item Questionnaire score ≥ 2. Data were analyzed using voxel-based morphometry (VBM), surface-based morphometry analysis (SBM), and logistic regression models.

**Results:**

SCD was defined in 243 persons and MCI in 246 individuals. The VBM analysis showed that MCI (vs. normal cognition) was significantly associated with reduced GMV in brain regions such as the bilateral parahippocampus, bilateral hippocampus, and bilateral fusiform (P < 0.05), but SCD exhibited no significant differences with normal cognition in GMV (P > 0.05). The ROI-wise SBM analysis revealed that SCD was significantly associated with cortical thinning in the right paracentral sulcus, left caudal middle frontal gyrus, and left entorhinal cortex (P < 0.05) and that MCI was significantly associated with cortical thinning in the left temporal lobe, left frontal lobe, bilateral parietal lobe and bilateral fusiform (P < 0.05).

**Conclusions:**

The brain regions with reduced GMV or cortical thickness in older adults gradually expand from normal cognition through SCD to MCI, suggesting that characterizing structural brain alterations may help define the cognitive spectrum at the pre-dementia phase. These findings have potential implications for understanding the neuropathological process of cognitive deterioration in aging.

## Highlights

• Structural brain alterations were analyzed using VBM and SBM among rural-dwelling older adults in China.
• Reduced cortical thickness was detected in six clusters of brain regions for MCI and three clusters for SCD.
• MCI, but not SCD, was associated with reduced GMV in four clusters of brain regions.
• The brain regions with reduced GMV or cortical thickness gradually expand from normal cognition through SCD to MCI.

## 1 Introduction

Mild cognitive impairment (MCI) is referred to as the point where cognitive impairment exists but is not severe enough to meet the diagnosis of dementia (“2023 Alzheimer's disease facts and figures,” 2023). Subjective cognitive decline (SCD) represents an earlier stage than MCI when people complain about their memory but show no evidence of objective cognitive impairment on the standardized neuropsychological testing and daily function (Jessen et al., 2020). SCD and MCI are considered transitional stages between normal cognitive aging and dementia (Gauthier et al., 2006, Jessen et al., 2020). A sizable majority of individuals with SCD may progress to MCI and then to Alzheimer’s dementia (AD), especially when co-existing with neuroimaging signs of AD (Sun, Yang, Lin, & Han, 2015).

Neuroimaging techniques with high spatial resolution, such as structural brain magnetic resonance imaging (MRI), enable accurate *in vivo* detection of subtle changes in brain structure of individuals with pre-AD (Rivas-Fernández et al., 2023). Thus, brain MRI scans may provide reliable imaging biomarkers to characterize subtle alterations in brain structure that can be used to define and differentiate individuals at pre-AD phases, which may facilitate early detection and therapeutic and preventive interventions (Young et al., 2020).

The whole-brain voxel-based comparison of grey matter volume (GMV) and the cortical surface-based comparison of cortical thickness are the two most common morphometric imaging analysis methods (Arrondo, Elía-Zudaire, Martí-Andrés, Fernández-Seara, & Riverol, 2022). Using the voxel-based morphometry (VBM) and the surface-based morphometry (SBM) analysis approaches, previous studies have reported structural brain alterations related to SCD and MCI. For example. a clinic-based study in China suggested that MCI was associated with grey matter atrophy in brain regions of the entorhinal cortex, frontal cortex, bilateral frontotemporal lobes, and postcentral gyrus (Huang et al., 2023). The multicenter clinic-based study revealed that thinner cortical thickness in patients with MCI was mainly confined to the bilateral parietal, frontal, temporal, and left precentral gyrus (Z. Ma et al., 2020). In additional, clinic-based studies also indicated that patients with SCD had decreased GMV in the bilateral entorhinal cortex, amygdala, hippocampus, parahippocampus, and left caudate nucleus regions (Chen et al., 2022) and thinner cortical thickness in the entorhinal cortex, perirhinal cortex, and medial orbitofrontal cortex (Fan et al., 2018). Of note, the large majority of previous studies have been conducted in clinical settings, where the findings may not be generalizable to the community-dwelling older adults.

Therefore, in this population-based MRI study of older adults who were living in rural communities in China, we used the VBM and SBM methods to characterize structural brain alterations (e.g., GMV and cortical thickness) associated with SCD and MCI.

## 2 Materials and methods

### 2.1 Study design and participants

**Fig. 1:**
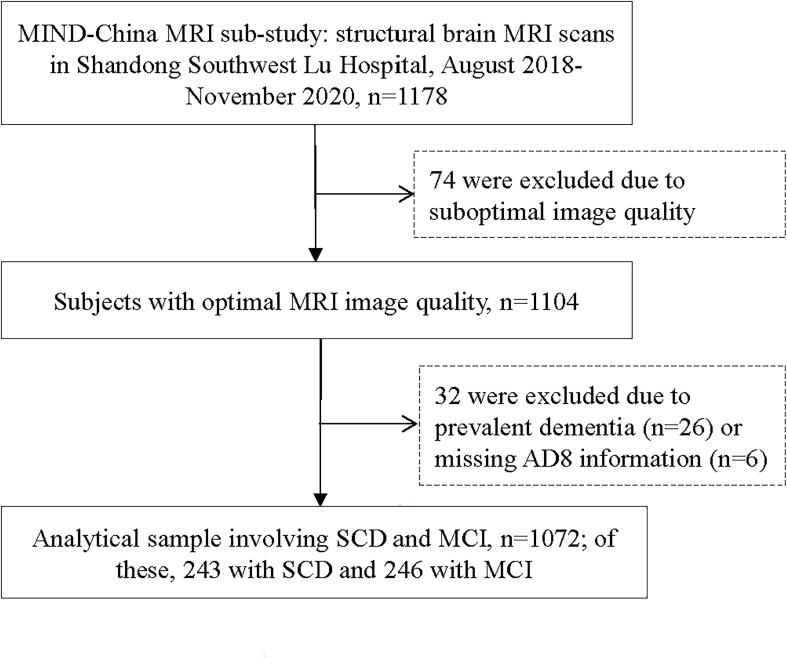
Flowchart of the study participants. Abbreviations: MIND-China, Multimodal Interventions to Delay Dementia and Disability in Rural China; MRI, magnetic resonance imaging; AD8, Ascertain Dementia 8-item Questionnaire; SCD, subjective cognitive decline; MCI, mild cognitive impairment.

This population-based cross-sectional study used data from the baseline assessments of the MRI Sub-study of the Multimodal Interventions to Delay Dementia and Disability in Rural China (MIND-China) study, as previously reported (Cong et al., 2023, Wang et al., 2022). In brief, the MIND-China study targeted people who were aged 60 years and older and living in the rural communities (52 villages) of Yanlou Town, Yanggu County in western Shandong Province. In March-September 2018, 5765 participants were examined for MIND-China, in which participants in the MRI sub-study were recruited from the 26 villages that were randomly selected from all the 52 villages. From August 2018 to November 2020, a subsample of 1178 individuals undertook structural brain MRI scans in Southwestern Lu Hospital. Of these, 106 persons were excluded due to suboptimal image quality (n = 74), dementia (n = 26), or missing information on the self-rated Ascertain Dementia 8-item Questionnaire (AD8) (n = 6), leaving 1072 participants for the current analysis. Fig. 1 shows the flowchart of the study participants (Fig. 1).

The protocol of the MIND-China study and the MRI sub-study was approved by the Ethics Committee of Shandong Provincial Hospital in Jinan, Shandong. Written informed consent was obtained from all participants. The MIND-China study was registered in the Chinese Clinical Trial Registry (registration No.: ChiCTR1800017758).

### 2.2 Data collection and assessments

In March-September 2018, data were collected by trained staff via face-to-face interviews, clinical examinations, neuropsychological tests, and laboratory tests following a standard questionnaire, as previously reported (Wang et al., 2022). In brief, we collected data on sociodemographic factors (e.g., age, sex, and years of education), lifestyle factors (e.g., smoking, alcohol drinking, and physical exercise), medical history (e.g., hypertension, diabetes, and dyslipidemia, ischemic heart disease, and stroke), and use of medications. All medications were classified and coded according to the Anatomical Therapeutic Chemical (ATC) classification system, as previously reported (Cong et al., 2020). Hypertension was defined as systolic pressure ≥ 140 mmHg or diastolic pressure ≥ 90 mmHg or current use of any antihypertensive drugs. Diabetes was defined as fasting blood glucose ≥ 7.0 mmol/L or self-reported physician diagnosis of diabetes or current use of antidiabetic medicine. Hyperlipidemia was defined as total serum cholesterol ≥ 6.2 mmol/L or triglyceride ≥ 2.3 mmol/L, or low-density lipoprotein cholesterol ≥ 4.1 mmol/L, or high-density lipoprotein cholesterol < 1.0 mmol/L, or use of hypolipidemic agents. Ischemic heart disease was identified by the examining physician according to self-report history of myocardial infarction, angina pectoris, coronary intervention, or pathologic Q waves on electrocardiogram. Stroke was ascertained according to self-reported history of stroke and neurological examination. The *APOE* genotype was determined using multiplex polymerase chain reaction amplification, as described in previous studies (Liang et al., 2022) and dichotomized into carriers and non-carriers of the *APOE* ε4 allele. The Mini-Mental State Examination (MMSE) and the self-rated AD8 were used to assess global cognitive function.

### 2.3 Diagnosis of MCI and SCD

Cognitive function was assessed using a neuropsychological test battery, as previously described (Cong et al., 2023). In brief, the function of following four cognitive domains was assessed: memory function (the Auditory Verbal Learning Test immediate recall, the long-delayed free recall, and the long-delayed recognition), verbal fluency (the Verbal Fluency Test of animal, fruit, and vegetable categories), attention (the Trail Making Test-A and Digit Span Forward test), and executive function (the Trail Making Test-B and Digit Span Backward test). Each of the raw test scores was standardized into z score using the mean and standard deviation (SD), derived from all participants who were free from dementia. Then, we generated the composite z score for each of the cognitive domains by averaging the z scores of all the tests for that domain. We used the Chinese version of activities of daily living to evaluate self-care and instrumental activities of daily living (ADLs). Dementia was clinically diagnosed according to the *Diagnostic and Statistical Manual of Mental Disorders*, Fourth Edition (DSM-IV), criteria, following a three-step diagnostic procedure, as previously reported (Wang et al. 2022). Among dementia-free participants, those with a cognitive z-score of 1.0 SD or more below the mean score for their specific age and education group in any of the four cognitive domains were classified as having objective cognitive impairment. However, the final judgement on objective cognitive impairment was made based on neuropsychological tests and a consensus among neurologists while taking into account the participants’ education, occupational history, presence of visual or hearing impairments, and other relevant factors.

We defined MCI according to the Petersen’s criteria that were operationalize in the way similar to that used in the Mayo Clinic Study of Aging (Petersen et al., 2010). The criteria are as follows: (1) cognitive concerns raised by the subject (based on responses to three questions regarding memory problems), an informant, or a physician (CDR ≥ 0.5); (2) objective cognitive impairment evident in at least one of the four cognitive domains (from the cognitive test battery); (3) essentially preserved daily activities functioning (from ADLs); and (4) absence of dementia (based on DSM-IV criteria).

We defined SCD following the principles outlined in the SCD-I recommendations (Jessen et al., 2014): (1) A consistent self-reported decline in cognitive ability compared to a previously normal cognitive status and (2) normal performance on standardized cognitive tests, adjusted for age, gender, and education, which are utilized to classify MCI or prodromal AD. Participants with MCI, prodromal AD, dementia, major psychiatric disorders, neurological diseases (apart from AD), medical disorders, and other systemic diseases that could cause cognitive impairment were excluded. The self-rated AD8(Galvin et al., 2005) was used to identify SCD among participants who were free of MCI and dementia. The AD8 test consists of eight items assessing changes in memory, problem-solving ability, orientation, and daily activities. The participants were asked to report any changes in their self-perceived cognitive issues that might have occurred over the past year compared with their previously normal cognitive function. The AD8 score was generated by counting the number of items marked as “Yes, a change”(range: 0–8). A higher AD8 score reflects poorer subjective cognitive function. Participants with an AD8 score of 2 or higher and no objective cognitive impairment were considered to have SCD.

### 2.4 MRI acquisition

From August 2018 to October 2020, all eligible participants were scanned on a Philips Ingenia 3.0 T MR System (Philips Healthcare, Best, The Netherlands) at the Southwestern Lu Hospital, as previously reported (Song et al., 2023, Wang et al., 2022). A comfortable foam padding was used to stabilize the head and minimize movement, and earplugs to reduce the scanner noise. The MRI scan parameters for the 3D-T1-weighted structural images were as follows: repetition time (TR) = 8.3 ms; echo time (TE) = 3.8 ms; field of view (FOV) = 240 × 219 mm^2^; acquisition matrix = 240 × 219; flip angle (FA) = 8◦; and slice thickness = 1 mm.

### 2.5 Voxel-based morphometry (VBM)

The VBM analysis was performed with the Computational Anatomy Toolbox (CAT 12; https://dbm.neuro.uni-jena.de/cat.html), an extension for the Statistical Parametric Mapping 12 (SPM12; https://www.fil.ion.ucl.ac.uk/spm/software/spm12/), on the platform of MATLAB R2016b. (i) All the 3D T1-weighted images were corrected for bias-field inhomogeneities. (ii) The high-resolution T1-images were segmented into different types of tissue, including grey matter (GM), white matter (WM), and cerebrospinal fluid (CSF). (iii) A customized, study-specific GM template in standard space was created with the Diffeomorphic Anatomical Registration using Exponentiated Lie (DARTEL) toolbox. (iv) GM probability maps were warped to the customized template and affine registered to the standard space (Montreal Neurological Institute space). (v) The nonlinear determinants derived from the spatial normalization are multiplied by the GM probability maps to obtain the GMV map. (vi) A 6-mm full-width-at-half-maximum (FWHM) Gaussian kernel was used for spatial smoothing of the GMV images. The smoothed GMV images were finally used in the following statistical analysis.

### 2.6 Surface-based morphometry (SBM)

The cortical thickness was measured using the CAT12 toolbox, which runs within SPM12. (i) These brain images were initially segmented into different types of tissue, including GM, WM, and CSF. (ii) The WM surface and pial surface were extracted, and the outer shell layer wrapping the pial surface was reconstructed. (iii) The cortical thickness was estimated by calculating the distance between the pial and WM surfaces. (iv) Surface reconstruction was performed in the right and left hemispheres separately by using a projection-based thickness calculation method (Dahnke, Yotter, & Gaser, 2013). (v) Topological correction and spherical mapping for inter-subject alignment and spherical registration were performed (Yotter et al., 2011, Yotter et al., 2011). (vi) An adapted DARTEL algorithm was then applied to the surface for spherical registration. Hemisphere meshes were merged and resampled to a template space (Glasser et al., 2013). (vii) Finally, the cortical thickness images were smoothed with a 15-mm FWHM Gaussian kernel. The smoothed images were finally used in the following statistical analysis.

### 2.7 Statistical analysis

To examine between-group (MCI or SCD vs. normal cognition and MCI vs. SCD) differences in GMV, we applied two-sample *t*-test using the SPM statistical software package in a voxel-wise manner, controlling for age, sex, and education. Multiple comparisons were corrected using the cluster-level family-wise error (FWE) method, with a voxel threshold being P < 0.001 and FWE-corrected P < 0.05. The GMV of each of those brain regions that showed between-group statistical difference was extracted using the DPABI toolbox. The logistic regression models were used to analyze associations of cognitive outcomes with regional GMV. The locations of the brain regions were defined based on the Automated Anatomical Labeling (AAL) atlas.

We conducted an ROI-wise SBM analysis to identify between-group differences in cortical thickness. In the calculations, we adopted the Desikan–Killiany atlas to subdivide each hemisphere into 34 ROIs, which contain information about either gyral or sulci structures in the human brain, and thus a total 68 ROIs for the whole cortex. Regional cortical thickness was compared between MCI and normal cognition, SCD and normal cognition, and SCD and MCI. Then, the two-sample *t*-test was conducted to evaluate between-group differences in cortical thickness implemented in the SPM statistical software package, controlling for age, sex, and education. Holm-Bonferroni correction was used for multiple comparison correction to control type I error (p < 0.05). The cortical thickness of each of those brain regions that showed between-group statistical difference was extracted using the CAT12 toolbox. The logistic regression models were used to analyze associations of cognitive status with regional cortical thickness. The locations of the brain regions were defined based on the Desikan–Killiany atlas.

We presented mean (standard deviation, SD) for continuous variables and frequencies (%) for categorical variables. Characteristics of the study participants by cognitive status were compared using nonparametric Kruskal–Wallis H test of variance for continuous variables and chi-square tests for categorical variables. We used binary logistic regression models to examine the associations of MCI with regional GMV and cortical thickness. We reported the main results from two models: model 1 was adjusted for age, sex, education, and if the analysis involved regional GMV, for total intracranial volume (ICV); and model 2 was additionally adjusted for ever smoking, alcohol intake, physical exercise, hypertension, diabetes, dyslipidemia, ischemic heart disease, stroke, and *APOE* genotype. IBM SPSS Statistics 26.0 for Windows (IBM Corp., Armonk, NY) was used for all other analyses.

## 3 Results

### 3.1 Characteristics of the study participants

Of the 1702 participants, 243 (14.28 %) were diagnosed with SCD and 246 (14.45 %) with MCI. The mean age of all participants was 69.50 years (SD = 4.33; age range 60–88 years), 58.12 % were women, and 33.96 % had no formal school education. There were statistically significant differences in mean age and the distributions of sex, education, smoking, alcohol intake, ischemic heart disease, and stroke among the three groups of people with SCD, MCI, and normal cognition (P < 0.05), but the three groups did not differ significantly in the distributions of occupation, physical exercise, body mass index (BMI), hypertension, diabetes, hyperlipidemia, and *APOE* ε4 status (Table 1).

**Table 1:** Characteristics of the study participants in the total sample and by cognitive status.

	Total sample	Cognitive status			
Characteristics	(n = 1072)	Normal cognition (n = 583)	SCD (n = 243)	MCI (n = 246)	P-value
**Age (years), mean (SD)**	69.50(4.33)	68.99(4.27)	69.88(4.36)	70.33(4.28)	<0.001
**Female sex, n (%)**	623(58.12)	303(51.97)	150(61.72)	170(69.11)	<0.001
**Education, n (%)**					<0.001
No formal education	364(33.96)	155(26.59)	96(39.51)	113(45.93)	
Primary school	485(45.24)	275(47.17)	103(42.39)	107(43.50)	
Middle school or above	223(20.80)	153(26.24)	44(18.11)	26(10.57)	
**Occupation, n (%)**					0.16
Farmer	935(87.46)	499(85.89)	211(86.83)	225(91.84)	
Non-farmer	134(12.54)	82(14.11)	32(13.17)	20(8.16)	
**Physical exercise, n (%)**					0.62
Weekly	218(20.34)	117(20.07)	46(18.93)	55(22.36)	
Less than weekly	854(79.66)	466(79.93)	197(81.07)	191(77.64)	
**BMI (kg/m^2^), mean (SD)**	25.10(3.53)	25.06(3.57)	25.24(3.50)	24.89(3.45)	0.54
**Ever smoking, n (%)**	388(36.19)	240(41.17)	86(35.39)	62(25.20)	<0.001
**Ever alcohol intake, n (%)**	361(34.06)	225(39.06)	81(33.75)	55(22.54)	<0.001
**Clinical conditions, n (%)**					
Hypertension	714(67.30)	385(66.84)	153(62.96)	176(72.72)	0.07
Diabetes mellitus	156(14.55)	90(15.44)	38(15.64)	28(11.38)	0.27
Hyperlipidemia	258(24.07)	139(23.84)	59(24.28)	60(24.39)	0.98
Ischemic heart disease	202(18.84)	96(16.47)	62(25.57)	44(17.89)	0.01
Stroke	125(13.48)	51(10.43)	38(17.67)	36(16.14)	0.01
***APOE* ε4 allele, n (%)**	158(15.19)	88(15.38)	33(14.29)	37(15.61)	0.85

Abbreviations: *APOE*, apolipoprotein E gene; BMI, body mass index; SD, standard deviation; SCD, subjective cognitive decline; MCI, mild cognitive impairment. Information was missing in 3 participants for occupation, 5 for BMI, 12 for alcohol consumption, 11 for hypertension, 139 for stroke, and 34 for *APOE* genotype.

### 3.2 Association of MCI with GMV clusters: VBM analysis

**Fig. 2:**
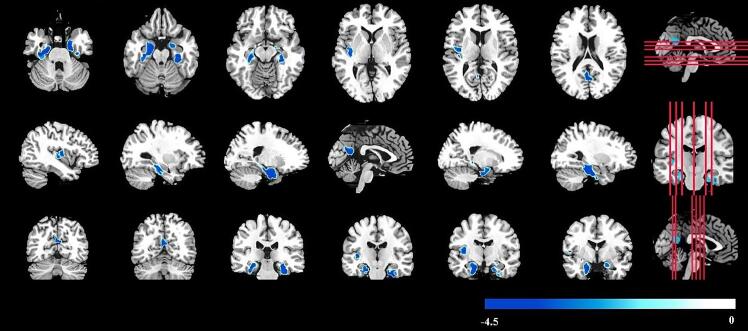
Comparison of whole-brain voxel-wise grey matter volume between normal cognition and mild cognitive impairment after cluster-level FWE correction. Note: Compared to individuals with normal cognition, people with mild cognitive impairment showed reduced grey matter volume mainly in the bilateral parahippocampus, bilateral hippocampus, bilateral fusiform, left precuneus, and left insula (FWE corrected p < 0.05 at cluster level). The color bar indicates the voxel-wise T-value.

The whole brain voxel-wise comparison analysis showed significant differences in four clusters of GMV between the MCI and normal cognition groups (Fig. 2). Overall, MCI was significantly associated with smaller GMV in the whole brain. Specifically, MCI (vs. normal cognition) was significantly associated with reduced GMV of four clusters that encompassed the bilateral parahippocampus, bilateral hippocampus, bilateral fusiform, left precuneus, and left insula (Table 2). The largest cluster of brain regions was located mainly in the right parahippocampus, with 44.98 % voxels in the right parahippocampus, 31.91 % voxels in the right fusiform, and 11.59 % voxels in the right hippocampus. The second largest cluster of brain regions was located mainly in the left parahippocampus, with 40.93 % voxels in the left parahippocampus, 32.16 % voxels in the left hippocampus, and 13.40 % voxels in the left fusiform.

**Table 2:** Characteristics of the four clusters of brain regions with greater grey matter volumes in people with normal cognition than participants with mild cognitive impairment.

Characteristics	Cluster 1	Cluster 2	Cluster 3	Cluster 4
Co-ordinates of peak-voxel	25.5, −16.5, −28.5	−21, −10.5, −30	−39, −19.5, 4.5	0, −61.5, 16.5
Peak T value	4.57	4.71	3.98	4.54
Cluster size	1545	1642	515	538
Overlap of atlas region	ParaHippocampal_R (44.98 %)	ParaHippocampal_L (40.93 %)	Insula_L (52.62 %)	Precuneus_L (51.86 %)
	Fusiform_R (31.91 %)	Hippocampus_L (32.16 %)	Rolandic_Oper_L (23.69 %)	Cingulum_Post_L (12.08 %)
	Hippocampus_R (11.59 %)	Fusiform_L (13.40 %)	Heschl_L (17.86 %)	Calcarine_R (10.41 %)
	Amygdala_R (2.14 %)	Amygdala_L (8.53 %)	Temporal_Sup_L (3.69 %)	Precuneus_R (9.48 %)
	Cerebelum_4_5_R (1.42 %)	Temporal_Pole_Mid_L (0.12 %)		Cuneus_L (6.88 %)

Note: ParaHippocampal_R, Right Parahippocampus; Fusiform_R, Right Fusiform; Hippocampus_R, Right Hippocampus; Amygdala_R, Right Amygdala; Cerebelum_4_5_R, Right Cerebellum 4–5; ParaHippocampal_L, Left Parahippocampus; Hippocampus_L, Left Hippocampus; Fusiform_L, Left Fusiform; Amygdala_L, Left Amygdala; Temporal_Pole_Mid_L, Left Middle Temporal Pole; Insula_L, Left Insula; Rolandic_Oper_L, Right Rolandic Operculum; Heschl_L, Left Heschl; Temporal_Sup_L, Left Superior Temporal gyrus; Precuneus_L, Left Precuneus; Cingulum_Post_L, Left Posterior Cingulate; Calcarine_R, Right Calcarine; Precuneus_R, Right Precuneus; Cuneus_L, Left Cuneus.

### 3.3 Associations of MCI with regional GMV: Logistic regression analysis

Controlling for multiple confounders, reduced GMV in the bilateral parahippocampus, bilateral hippocampus, bilateral fusiform, left precuneus, left Rolandic operculum, left Heschl, and left posterior cingulate gyrus was significantly associated with increased likelihoods of MCI, even after false discovery rate (FDR) corrections for multiple comparisons (Table 3).

**Table 3:** Associations of mild cognitive impairment with regional grey matter volume.

Brain regions	Grey matter volume, mean (SD)		Odds ratio (95 % confidence interval), mild cognitive impairment			
	Normal cognition (n = 583)	MCI (n = 246)	Model 1	FDR-corrected *p-*value	Model 2	FDR-corrected *p-*value
ParaHippocampal_R	0.41(0.04)	0.39(0.04)	0.69 (0.56, 0.84)^***^	0.001	0.66 (0.53, 0.81)^***^	<0.001
Fusiform_R	0.39(0.04)	0.38(0.04)	0.69 (0.56, 0.85)^***^	0.001	0.68 (0.54, 0.84)^***^	<0.001
Hippocampus_R	0.38(0.04)	0.36(0.05)	0.76 (0.64, 0.91)^**^	0.004	0.74 (0.62, 0.89)^**^	0.002
ParaHippocampal_L	0.39(0.04)	0.37(0.04)	0.64 (0.52, 0.79)^***^	<0.001	0.63 (0.51, 0.77)^***^	<0.001
Hippocampus_L	0.38(0.04)	0.36(0.05)	0.74 (0.61, 0.88)^***^	0.002	0.72 (0.60, 0.87)^***^	<0.001
Fusiform_L	0.39(0.04)	0.37(0.04)	0.73 (0.60, 0.90)^**^	0.004	0.72 (0.58, 0.88)^**^	0.003
Insula_L	0.40(0.04)	0.39(0.04)	0.85 (0.71, 1.03)	0.093	0.85 (0.70, 1.02)	0.087
Rolandic_Oper_L	0.32(0.04)	0.30(0.04)	0.70 (0.58, 0.85)^***^	0.001	0.68 (0.56, 0.83)^***^	<0.001
Heschl_L	0.37(0.05)	0.34(0.05)	0.72 (0.60, 0.86)^***^	0.001	0.70 (0.59, 0.85)^***^	<0.001
Precuneus_L	0.31(0.03)	0.30(0.03)	0.69 (0.56, 0.85)^***^	0.001	0.67 (0.54, 0.83)^***^	<0.001
Cingulum_Post_L	0.30(0.04)	0.29(0.04)	0.77 (0.64, 0.93)^**^	0.006	0.76 (0.63, 0.91)^**^	0.004
Calcarine_R	0.29(0.04)	0.28(0.04)	0.85 (0.71, 1.02)	0.090	0.84 (0.69, 1.00)	0.061

Note: Data in Model 1 and Model 2 were odds ratio (95 % confidence interval) of MCI associated with per 1-SD increase in grey matter volume. Model 1 was controlled for age, sex, education, and total intracranial volume; model 2 was additionally adjusted for ever smoking, alcohol intake, physical exercise, hypertension, diabetes, dyslipidemia, ischemic heart disease, stroke, and *APOE* genotype. Individuals with normal cognition were used as reference group in the modeling analysis. MCI, mild cognitive impairment; ParaHippocampal_R, Right Parahippocampus; Fusiform_R, Right Fusiform; Hippocampus_R, Right Hippocampus; ParaHippocampal_L, Left Parahippocampus; Hippocampus_L, Left Hippocampus; Fusiform_L, Left Fusiform; Insula_L, Left Insula; Rolandic_Oper_L, Right Rolandic Operculum; Heschl_L, Left Heschl; Precuneus_L, Left Precuneus; Cingulum_Post_L, Left Posterior Cingulate gyrus; Calcarine_R, Right Calcarine. * p < 0.05, ^**^p < 0.01, ^***^p < 0.001.

### 3.4 Association of MCI and SCD with clusters of regional grey matter cortical thickness: SBM analysis

**Fig. 3:**
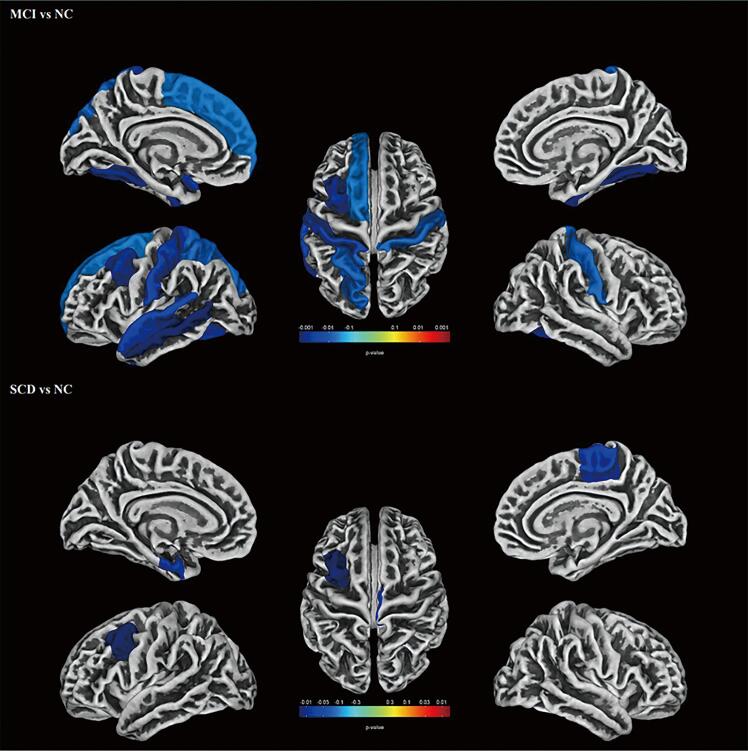
Comparison of whole-brain ROI-based cortical thickness between mild cognitive impairment and normal cognition, as well as between subjective cognitive decline and normal cognition after Holm-Bonferroni correction. Note: Compared to persons with normal cognition, individuals with mild cognitive impairment exhibited reduced cortical thickness mainly in the bilateral postcentral gyrus, bilateral fusiform gyrus, left superior parietal lobe, left superior frontal gyrus, left caudal middle frontal gyrus, left superior temporal gyrus and left middle temporal gyrus (Holm-Bonferroni correction, p < 0.05). People with subjective cognitive decline exhibited reduced cortical thickness mainly in the right paracentral sulcus, left caudal middle frontal gyrus, and left entorhinal cortex (Holm-Bonferroni correction, p < 0.05). The color bar indicates the P-value.

The ROI-wise SBM analysis revealed six clusters of brain regions that were significantly associated with MCI, and three clusters of brain regions that were significantly associated with SCD (Fig. 3). Overall, compared with people with normal cognition, those with MCI and SCD showed a thinner cortical thickness. The MCI-related six clusters of thinner cortical thickness encompassed the bilateral postcentral gyrus, bilateral fusiform gyrus, left superior parietal lobe, left superior frontal gyrus, left caudal middle frontal gyrus, left superior temporal gyrus, and left middle temporal gyrus (Table 4). The largest cluster of brain regions was located mainly in the left superior parietal lobe, with 52 % vertexes in the left superior parietal lobe and 48 % vertexes in the left postcentral gyrus, followed by the cluster of brain regions located mainly in the left superior frontal gyrus, with 75 % vertexes in the left superior frontal gyrus and 23 % vertexes in the left caudal middle frontal gyrus. The third largest cluster of brain regions was located mainly in the left superior temporal gyrus, with 62 % vertexes in the left superior temporal gyrus and 38 % vertexes in the left middle temporal gyrus. All voxels in the remaining three clusters were located in the right postcentral gyrus, left fusiform gyrus, and right fusiform gyrus, respectively. Reduced cortical thickness in people with SCD was observed in three clusters located in right paracentral sulcus, left caudal middle frontal gyrus, and left entorhinal cortex (Table 4). The largest cluster of brain regions was located mainly in the right paracentral sulcus, followed by the cluster of brain regions located mainly in the left caudal middle frontal gyrus. The smallest cluster of brain regions was located in the left entorhinal cortex.

**Table 4:** Characteristics of brain regions with reduced cortical thickness associated with mild cognitive impairment and subjective cognitive decline.

Characteristics	Cluster 1	Cluster 2	Cluster 3	Cluster 4	Cluster 5	Cluster 6
**Mild cognitive impairment**						
T-value	−3.29	−3.58	−3.78	−2.92	−3.61	−3.71
Cluster size	19,975	16,187	11,723	9138	4714	4661
Overlap of atlas region	Superiorparietal_L (52 %) Postcentral_L (48 %)	Superiorfrontal_L (75 %) Caudalmiddlefrontal_L (23 %) Frontalpole_L (2 %)	Superiortemporal_L (62 %) Middletemporal_L (38 %)	Postcentral_R (100 %)	Fusiform_L (100 %)	Fusiform_R (100 %)

**Subjective cognitive decline**						
T-value	−2.55	−2.95	−2.76	−	−	−
Cluster size	3831	3736	1087	−	−	−
Overlap of atlas region	Paracentral_R (100 %)	Caudalmiddlefrontal_L (100 %)	Entorhinal_L (100 %)	−	−	−

Note: Superiorparietal_L, left superior parietal lobe; Postcentral_L, left postcentral gyrus; Superiorfrontal_L, left superior frontal gyrus; Caudalmiddlefrontal_L, left caudal middle frontal gyrus; Frontalpole_L, left frontal pole; Superiortemporal_L, left superior temporal gyrus; Middletemporal_L, left middle temporal gyrus; Postcentral_R, right postcentral gyrus; Fusiform_L, left fusiform gyrus; Fusiform_R, right fusiform gyrus; Paracentral_R, right paracentral sulcus; Entorhinal_L, left entorhinal cortex.

### 3.5 Associations of MCI and SCD with regional grey matter cortical thickness: Logistic regression analysis

Controlling for potential confounding factors and even after FDR corrections, thinner cortical thickness of the brain regions in the bilateral postcentral gyrus, bilateral fusiform gyrus, left superior parietal lobe, left superior frontal gyrus, left caudal middle frontal gyrus, left superior temporal gyrus, and left middle temporal gyrus was significantly associated with increased likelihoods of MCI (Table 5). Similarly, controlling for all the examined potential confounders, thinner cortical thickness of the right paracentral sulcus, left caudal middle frontal gyrus, and left entorhinal cortex was significantly associated with an increased likelihood of SCD, even after FDR corrections (Table 5).

**Table 5:** Associations of subjective cognitive decline or mild cognitive impairment with regional grey matter cortical thickness.

Brain regions	Grey matter thickness (mm), mean (SD)		Odds ratio (95 % confidence interval), MCI or SCD			
	Normal cognition (n = 583)	MCI or SCD	Model 1	FDR corrected *p-*value	Model 2	FDR corrected *p-*value
**Mild cognitive impairment** (n = 246)						
Superiorparietal_L	2.24(0.11)	2.21(0.12)	0.77 (0.66, 0.90)^**^	0.002	0.77 (0.66, 0.90)^**^	0.002
Postcentral_L	2.03(0.13)	2.00(0.13)	0.74 (0.62, 0.88)^***^	0.001	0.74 (0.63, 0.89)^***^	0.002
Superiorfrontal_L	2.87(0.12)	2.83(0.15)	0.77 (0.65, 0.90)^**^	0.002	0.77 (0.65, 0.91)^**^	0.002
Caudalmiddlefrontal_L	2.70(0.13)	2.66(0.15)	0.74 (0.63, 0.87)^***^	0.001	0.74 (0.63, 0.87)^***^	0.001
Superiortemporal_L	2.69(0.13)	2.65(0.15)	0.74 (0.62, 0.87)^***^	0.001	0.73 (0.62, 0.87)^***^	0.001
Middletemporal_L	2.92(0.14)	2.87(0.16)	0.73 (0.62, 0.85)^***^	0.001	0.74 (0.62, 0.87)^***^	0.001
Postcentral_R	2.05(0.13)	2.03(0.13)	0.79 (0.66, 0.93)^**^	0.005	0.78 (0.66, 0.93)^**^	0.005
Fusiform_L	2.71(0.14)	2.67(0.16)	0.78 (0.66, 0.92)^**^	0.003	0.78 (0.66, 0.92)^**^	0.003
Fusiform_R	2.72(0.15)	2.68(0.15)	0.76 (0.65, 0.90)^**^	0.002	0.76 (0.64, 0.90)^**^	0.002

**Subjective cognitive decline** (n = 243)						
Paracentral_R	2.39(0.16)	2.35(0.16)	0.77 (0.66, 0.91)^**^	0.004	0.77 (0.65, 0.90)^**^	0.004
Caudalmiddlefrontal_L	2.70(0.13)	2.67(0.13)	0.83 (0.71, 0.97)*	0.019	0.82 (0.70, 0.96)*	0.020
Entorhinal_L	3.68(0.41)	3.60(0.41)	0.83 (0.71, 0.97)*	0.019	0.84 (0.72, 0.98)*	0.032

Note: Data in Model 1 and Model 2 were odds ratio (95 % confidence interval) of SCD or MCI associated with per 1-SD increase in grey matter cortical thickness. Model 1 was controlled for age, sex, and education; model 2 was additionally adjusted for ever smoking, alcohol intake, physical exercise, hypertension, diabetes, dyslipidemia, ischemic heart disease, stroke, and *APOE* genotype. Individuals with normal cognition were used as reference group in the modeling analysis. SCD, Subjective cognitive decline; MCI, mild cognitive impairment; Superiorparietal_L, left superior parietal lobe; Postcentral_L, left postcentral gyrus; Superiorfrontal_L, left superior frontal gyrus; Caudalmiddlefrontal_L, left caudal middle frontal gyrus; Superiortemporal_L, left superior temporal gyrus; Middletemporal_L, left middle temporal gyrus; Postcentral_R, right postcentral gyrus; Fusiform_L, left fusiform gyrus; Fusiform_R, right fusiform gyrus; Paracentral_R, right paracentral sulcus; Entorhinal_L, left entorhinal cortex. * p < 0.05, ^**^p < 0.01, ^***^p < 0.001.

### 3.6 GMV differences between MCI and SCD: VBM analysis

The whole brain voxel-wise comparison analysis showed significant differences in two clusters of GMV between the MCI and SCD groups (Fig. S1). Overall, compared with individuals with SCD, those with MCI showed smaller GMV in the whole brain. There were significant differences between the MCI and SCD groups in GMV of two clusters that encompassed the bilateral hippocampus, bilateral parahippocampus, bilateral amygdala, bilateral fusiform, left middle temporal pole, left superior temporal pole, and right cerebellum (Table S1). The largest cluster of brain regions was located mainly in the right parahippocampus, with 50.39 % voxels being in the right parahippocampus, 25.48 % voxels in the right hippocampus, and 15.06 % voxels in the right fusiform. The second largest cluster of brain regions was located mainly in the left parahippocampus, with 51.36 % voxels being in the left parahippocampus, 22.33 % voxels in the left hippocampus, and 13.88 % voxels in the left amygdala.

## 4 Discussion

In this population-based study of rural-dwelling older adults in China, using VBM and SBM analysis approaches, we sought to characterize structural brain alterations associated with MCI and SCD by focusing on cortical thickness and GMV. Overall, brain regions with reduced cortical thickness and GMV showed a gradual expansion across the cognitive spectrum from normal cognition through SCD to MCI. Specifically, we found that SCD was associated with thinner cortical thickness mainly in the right paracentral sulcus, left caudal middle frontal gyrus, and left entorhinal cortex, although we found no evidence that SCD was associated with reduced GMV in any of the examined brain areas in rural older adults. Furthermore, MCI was associated with reduced GMV predominantly in the bilateral parahippocampus, bilateral hippocampus, and bilateral fusiform, and thinner cortical thickness in the left caudal middle frontal gyrus, left superior parietal lobe, left superior frontal gyrus, left superior temporal gyrus, left middle temporal gyrus, bilateral fusiform, and the bilateral postcentral gyrus. Finally, MCI (vs. SCD) was associated with reduced GMV mainly in the bilateral hippocampus, parahippocampus, amygdala, and fusiform, but not with thinner cortical thickness in any of the examined brain regions.

Previous studies have examined alterations of regional GMV related to MCI mostly in the clinical settings. For instance, a clinic-based study of patients with MCI indicated that the atrophic GM regions in patients with MCI involved the bilateral medial temporal areas, left thalamus, and left precuneus (Han et al., 2021). In addition, a *meta*-analysis of 22 case-control studies showed volume reduction in the bilateral amygdala and hippocampus associated with MCI (Mieling, Meier, & Bunzeck, 2023). Our population-based study of rural older adults revealed that structural brain alteration in MCI was characterized by reductions in four clusters of brain regions covering parts of the bilateral parahippocampus, bilateral hippocampus, bilateral fusiform, and left precuneus, which was overall consistent with previous reports from the clinic-based studies. Furthermore, we detected additional brain regions (e.g., the right Rolandic operculum and left Heschel) where reduced GMV was associated with MCI. The left Heschl’s gyrus is responsible for auditory processing (Slade, Plack, & Nuttall, 2020), whereas the left Rolandic operculum controls the movement of articulatory organs during language production (Behroozmand et al., 2015). A prolonged lack of auditory stimulation (e.g., music or conversation) and motor coordination training (e.g., pronunciation, chewing, or swallowing) lead to a decline in the activity frequency of these brain regions among our rural participants. As a result, neuronal connectivity and plasticity may decrease, and lead to functional and structural degradation of these brain regions. However, we found no evidence for reduced GMV associated with SCD compared with normal cognition. While this was largely consistent with several previous reports (Kawagoe et al., 2019, Parker et al., 2020), some clinic-based studies did show that SCD was associated with reductions in GMV of the bilateral medial temporal, frontal gyrus, and occipital gyrus (Rivas-Fernández et al., 2023, Riverol et al., 2024). The discrepant findings across studies might be partly due to differences in the study settings (e.g., volunteers vs. memory clinics), demographic characteristics of the study samples (e.g., rural residents with no or limited education vs. highly educated urban residents), and the analysis methods (e.g., whole-brain regression analysis vs. seed-based analysis). Therefore, GMV alterations of older adults with SCD should be further characterized in large-scale population-based studies. Overall, our study showed that MCI, but not SCD, was associated with reduced GMV in several brain regions. This could be partly explained by the view that neuronal dysfunction may precede neuronal atrophy in the brain aging and neurodegenerative process (Jack et al., 2013). Alternatively, synaptic dysfunction may already cause subtle memory and cognitive lapses before atrophy can be detectable.

We detected six clusters of brain regions with reduced cortical thickness associated with MCI, which were located in the bilateral postcentral gyrus, bilateral fusiform gyrus, left caudal middle frontal gyrus, left superior parietal lobe, left superior frontal gyrus, left superior temporal gyrus, and left middle temporal gyrus. Data from the Mayo Clinic Study of Aging also suggested that people with MCI had thinner cortical thickness in the entorhinal cortex, middle/superior temporal gyrus, middle frontal gyrus, and superior frontal gyrus (Machulda et al., 2020), which is in line with the findings from our study. Additionally, with regard to structural brain alterations related to SCD, three clusters of brain regions with reduced cortical thickness were detected, located in the right paracentral sulcus, left caudal middle frontal gyrus, and left entorhinal cortex, which was consistent with the results from a register-based study of cognitively normal adults at risk for Alzheimer’s dementia (Schultz et al., 2015). However, a community-based study in Canada did show no significant differences in cortical thickness between SCD and normal cognition (Marcotte, Potvin, Collins, Rheault, & Duchesne, 2019). Differences in the methods for defining SCD (e.g., self-reported AD8 score ≥ 2 vs. a single question about subjective cognitive concerns) and the analytical methods (whole-brain regression analysis vs. seed-based correlation analysis) might partly contribute to the discrepant findings across studies. Taken together, we detected six clusters of brain regions with reduced cortical thickness associated with MCI and three clusters of brain regions with reduced cortical thickness in individuals with SCD. This implies that subtle changes in brain structure in older adults with SCD may begin typically from aging-sensitive regions like the entorhinal cortex and frontal lobes. Along with progression from SCD to MCI, cortical atrophy or reduction in cortical thickness spreads more widely to multiple brain regions across the entorhinal cortex, other parts of medial temporal lobe, frontal gyrus, and parietal lobe. These findings support the view that areas of brain atrophy in older people are expanded along with cognitive deterioration from normal cognition through SCD to MCI (Yue et al., 2018). However, it was worth noting that certain brain regions (e.g., right paracentral and left entorhinal cortices) exhibited reduced cortical thickness only in SCD but not in MCI. This may be due to the neural networks of the brain being reorganized and adjusted during the neurodegenerative process (D'Amelio & Rossini, 2012). As cognitive function declines, the memory-related neural network (e.g., default mode network) may increase connectivity to compensate for functional decline in areas (e.g., left entorhinal cortex)(Zhang et al., 2012). This compensatory mechanism may mask cortical thinning in these brain regions, while other areas may present more prominent structural degeneration. This suggests the complex relationship of dynamic changes in neural networks with cognitive deterioration that involves not only cerebral atrophic process but also intricate compensatory adjustments and neuroplasticity (Hartwigsen, 2018).

We characterized subtle alterations of SCD and MCI in regional grey matter using two analytical methods (e.g., SBM and VBM analyses). The ROI-wise SBM analysis suggested that the cortical thickness in persons with SCD was reduced in the right paracentral sulcus, left caudal middle frontal gyrus, and left entorhinal cortex. However, the VBM analysis did not detect any significant alterations in GMV associated with SCD. This was in line with a study of individuals with motoric cognitive risk syndrome that applied both SBM and VBM methods (Blumen et al., 2021). Although both VBM and SBM analyses adopt similar preprocessing steps, the two methods utilize different strategies in aligning the image to the template. The VBM analysis employs voxel-based registration to compensate for individual variability, but it is relatively susceptible to registration artifacts caused by global volume changes. The high variability in folding patterns across individuals may further diminish the accuracy of alignment in VBM (Anticevic et al., 2008). In contrast, the SBM analysis uses an alternative approach for registration by matching the gyral and sulcal geometry to an inflated spherical atlas (Winkler et al., 2010). The SBM analysis significantly reduces the potential misalignment caused by complex folding patterns as well as global volumetric changes. Additionally, cortical thickness measured by the SMB method directly reflects its own grey matter thickness, whereas the VBM analysis provides a combined measure of cortical surface area, cortical folding, and grey matter thickness. Hence, compared to VBM analysis, analysis of cortical thickness with SBM appears to be more sensitive in detecting subtle structural brain alterations (Winkler et al., 2010).

A neuropathological study revealed an increased burden of neuritic plaques and neurofibrillary tangles in the frontal, parietal, and temporal lobes, and in the hippocampal and entorhinal regions in patients with MCI (Dugger et al., 2015). In addition, the fluorodeoxyglucose-positron emission tomography (FDG-PET) study of patients with MCI showed cerebral hypometabolism in the medial temporal lobe, precuneus, and frontolimbic regions (Cerami et al., 2018, Ma et al., 2018, Riederer et al., 2018). Similarly, the FDG-PET imaging studies revealed that patients with SCD had hypometabolism in the medial temporal and parietal regions, including the precuneus (Dong et al., 2021, Scheef et al., 2012). Taken together, evidence from the neuropathological and metabolic imaging studies supports the characteristics of structural brain alterations for MCI and SCD identified in our population-based study.

Our population-based study engaged the rural-dwelling Chinese older adults who had no or very limited school education, a sociodemographic group that has so far received little attention from the research community. Our study does have limitations that deserve mentioning. First, the cross-sectional study might lead to an underestimation of the true association between brain structures and cognitive phenotypes due to selective survival. Furthermore, participants in MIND-China were recruited from only one rural area in western Shandong Province and the MRI subsample was relatively younger and healthier compared with the MIND-China total sample. This should be kept in mind when generalizing our research results to other populations.

## 5 Conclusion

In conclusion, our population-based study of rural-dwelling older adults in China revealed an expansion of brain regions with reduced GMV or cortical thickness across the cognitive spectrum from normal cognition through SCD to MCI. Specifically, subtle structural brain alterations for MCI were characterized by reduced GMV predominantly in the bilateral medial temporal lobe and bilateral fusiform, and thinner cortical thickness in the left frontal gyrus, bilateral parietal lobe, bilateral fusiform, and left temporal gyrus; the brain structures for SCD are featured with thinner cortical thickness mainly in the right paracentral sulcus, left caudal middle frontal gyrus, and left entorhinal cortex. These results have potential implications for defining the cognitive spectrum at the pre-dementia phases, for understanding the neuropathological process of cognitive deterioration in aging as well as for early detection of SCD and MCI in rural older residents. Future studies that involve urban older populations are warranted to bridge the knowledge gaps among geographically and socioeconomically diverse populations.

## CRediT authorship contribution statement

**Ziwei Chen:** Writing – review & editing, Writing – original draft, Visualization, Methodology, Investigation, Formal analysis, Data curation, Conceptualization. **Qianqian Xie:** Writing – review & editing, Methodology, Investigation, Data curation. **Jiafeng Wang:** Methodology, Investigation, Data curation. **Yan Wang:** Writing – review & editing, Investigation, Data curation. **Huisi Zhang:** Methodology, Investigation, Data curation. **Chunyan Li:** Investigation, Data curation. **Yongxiang Wang:** Project administration, Investigation, Data curation. **Lin Cong:** Project administration, Investigation, Data curation. **Shi Tang:** Project administration, Methodology, Data curation. **Tingting Hou:** Project administration, Investigation, Data curation. **Lin Song:** Project administration, Investigation, Data curation. **Yifeng Du:** Supervision, Resources, Project administration, Investigation, Funding acquisition, Data curation, Conceptualization. **Chengxuan Qiu:** Writing – review & editing, Project administration, Methodology, Investigation, Funding acquisition, Data curation, Conceptualization.

## Funding

The MIND-China Study was financially supported by grant from the STI2030-Major Project (grant no.: 2021ZD0201808 and 2022ZD0211600) and by additional grants from the National Key R&D Program of China (grant no.: 2017YFC1310100), the National Nature Science Foundation of China (grants no.: 82171175, 82011530139, and 82001120), the Nature Science Foundation of Shandong Province (grant no.: ZR2020QH098), the Academic Promotion Program of Shandong First Medical University (grants no.: 2019QL020 and 2020RC009), and the Taishan Scholar Program of Shandong Province (grants no.: ts20190977 and Tsqn201909182). C Qiu received grants from the Swedish Research Council (grants no.: 2017–05819 and 2020–01574) and the Swedish Foundation for International Cooperation in Research and Higher Education (STINT) (grant no.: CH2019-8320), Stockholm, Sweden. The funding agencies had no role in the study design, data collection, and analysis, the writing of this manuscript, and in the decision to submit the work for publication.

## Data Availability

The datasets used and/or analyzed during the current study are available from the corresponding author upon reasonable request
