# Supplementary material for: Mapping grey matter and cortical thickness alterations associated with subjective cognitive decline and mild cognitive impairment among rural-dwelling older adults in China: A population-based study
Source: Neuroimage Clin. 2024 Oct 28;44:103691. doi: 10.1016/j.nicl.2024.103691 (PMC11566878; doi:10.1016/j.nicl.2024.103691)
Supplement: Supplementary Data 1 [file mmc1.docx]

**Supplementary materials**

**Mapping grey matter and cortical thickness alterations associated with subjective cognitive decline and mild cognitive impairment among rural older adults in China: a population-based study**

Chen Z, et al.

**Contents**

**Fig. S1.** Comparison of whole-brain voxel-wise grey matter volume between participants with subjective cognitive decline and those with mild cognitive impairment after cluster-level FWE correction.

**Table S1.** Characteristics of the two clusters of brain regions with reduced grey matter volume in individuals with mild cognitive impairment than those with subjective cognitive decline.


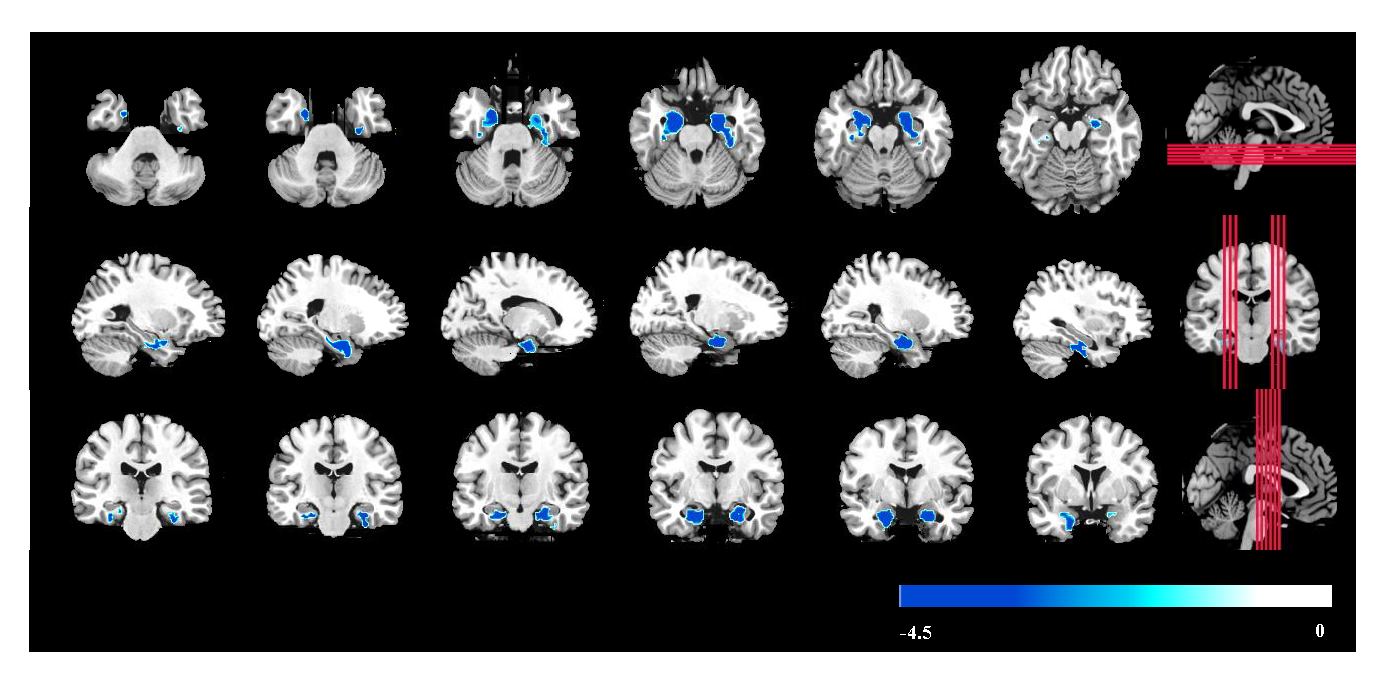


**Fig. S1.** Comparison of whole-brain voxel-wise grey matter volume between participants with subjective cognitive decline and those with mild cognitive impairment after cluster-level FWE correction.

Note: Compared to individuals with subjective cognitive decline, people with mild cognitive impairment showed reduced grey matter volume mainly in the bilateral hippocampus, bilateral parahippocampus, bilateral amygdala, and bilateral fusiform (FWE corrected p<0.05 at cluster level). The color bar indicates the voxel-wise T-value.

**Table S1.** Characteristics of the two clusters of brain regions with reduced grey matter volume in individuals with mild cognitive impairment than those with subjective cognitive decline.

| **Characteristics** | **Cluster 1** | **Cluster 2** |
| --- | --- | --- |
| Co-ordinates of peak-voxel | 31.5, -16.5, -25.5 | -30, -19.5, -24 |
| Peak T value | -4.48 | -4.26 |
| Cluster size | 1036 | 1030 |
| Overlap of atlas region | ParaHippocampal_R (50.39%) | ParaHippocampal_L (51.36%) |
|  | Hippocampus_R (25.48%) | Hippocampus_L (22.33%) |
|  | Fusiform_R (15.06%) | Amygdala_L (13.88%) |
|  | Amygdala_R (3.57%) | Fusiform_L (5.34%) |
|  | Cerebelum_4_5_R (0.29%) | Temporal_Pole_Mid_L (2.04%)  Temporal_Pole_Sup_L (0.87%) |

Note: Hippocampus_L, Left Hippocampus; Hippocampus_R, Right Hippocampus; ParaHippocampal_L, Left Parahippocampus; ParaHippocampal_R, Right Parahippocampus; Amygdala_L, Left Amygdala; Amygdala_R, Right Amygdala; Fusiform_L, Left Fusiform; Fusiform_R, Right Fusiform; Temporal_Pole_Mid_L, Left Middle Temporal Pole; Temporal_Pole_Sup_L, Left Superior Temporal Pole; Cerebelum_4_5_R, Right Cerebellum 4-5.
